# Brain perfusion during manic episode and at 6‐month follow‐up period in bipolar disorder patients: Correlation with cognitive functions

**DOI:** 10.1002/brb3.1615

**Published:** 2020-05-01

**Authors:** Maria Anayali Estudillo‐Guerra, Kevin Pacheco‐Barrios, Alejandra Cardenas‐Rojas, Gloria Adame‐Ocampo, Joan A. Camprodon, Leon Morales‐Quezada, Doris Gutiérrez‐Mora, Mónica Flores‐Ramos

**Affiliations:** ^1^ Clínica de Trastornos de Afecto National Institute of Psychiatry “Ramón de la Fuente Muñiz” México City México; ^2^ Department of Physical Medicine and Rehabilitation Spaulding Rehabilitation Hospital Harvard Medical School Boston MA USA; ^3^ Neuromodulation Center and Center for Clinical Research Learning Spaulding Rehabilitation Hospital and Massachusetts General Hospital Boston MA USA; ^4^ San Ignacio de Loyola University Lima Peru; ^5^ National Institute of Psychiatry “Ramón de la Fuente Muñiz” Servicio de Neuroimagen México City México; ^6^ Department of Psychiatry Laboratory for Neuropsychiatry and Neuromodulation Massachusetts General Hospital Harvard Medical School Boston MA USA; ^7^ National Council on Science and Technology, CONACYT México City México; ^8^ Teaching Department National Institute of Psychiatry “Ramón de la Fuente Muñiz” México City México

**Keywords:** bipolar disorder, emission‐computed, functional neuroimaging, single‐photon, tomography

## Abstract

**Background:**

Patterns of altered cerebral perfusion and cognitive dysfunction have been described in Bipolar Disorder (BD) acute episodes and euthymia. Knowledge of the relationship between cognitive function and perfusion in a manic state and status when followed up is still limited.

**Objective:**

To describe brain perfusion alterations and its relationship with cognitive impairment in patients with BD during manic episodes and after 6 months.

**Methods:**

Observational‐prospective study in 10 type I BD adults during moderate‐severe manic episodes. We assessed sociodemographic data and clinical variables as well as cognitive function through Screening for Cognitive Impairment in Psychiatry (SCIP‐S). Finally, we performed a Brain Perfusion SPECT using a Tc99m‐ethyl cysteine dimer.

**Results:**

During manic episodes, patients showed cognitive impairment with a mean SCIP‐S score of 63.8 ± 17.16. This was positively correlated with perfusion measured as relative reuptake index (RRI) at the right temporal pole (*ρ* = 0.65 *p* = .0435) and negatively correlated with right the orbitofrontal cortex (*ρ* = −0.70 *p* = .0077) and the right subgenual cingulate cortex (*ρ* = −0.70 *p* = .0256). Episode severity measured by the Young Mania Rating Scale (YMRS) positively correlated with RRI at the right temporal pole (*ρ* = 0.75, *p* = .01). At follow‐up, six patients were taking treatment and were euthymic, we found a negative correlation with the YMRS and RRI at the bilateral orbitofrontal cortex (*ρ* = −0.8827, *p* = .019). They did not show significant improvement in cognitive performance at SCIP‐S, and there was negative correlation with the following of the SCIP‐S subscales; processing speed with the bilateral dorsolateral prefrontal, the bilateral medial prefrontal, the left temporal pole cortex RRI, and verbal fluency with the bilateral anterior cingulate cortex RRI.

**Conclusion:**

Cognitive impairment was correlated with brain perfusion patterns at baseline and follow‐up. Large sample size studies with longer follow‐up are needed to describe the changes in perfusion and cognitive functions in BD.

## Graphical abstract

Observational‐prospective study in 10 type I BD adults during moderate‐severe manic episode that evaluates brain perfusion and cognition. Cognitive performance did not improve despite clinical improvement at follow‐up, and it was correlated to neocortical decreased perfusion. Clinical improvement was related to an increase in perfusion at orbitofrontal areas.

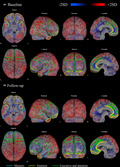

## 1 INTRODUCTION

Type I BD is a complex, chronic, and severe mental illness; it is characterized by at least one manic or mixed‐manic episode during the patient's lifetime (First, 2013). BD has a prevalence of 1% worldwide (Alonso et al., 2011), while 1.9% for Mexican population (Medina‐Mora, Borges, Benjet, Lara, & Berglund, 2007). This chronic disease is associated with decreased quality of life, as well as functional status (Bora, 2018). BD is related to a major risk of disability and suicide (Grande et al., 2013), (Michalak, Yatham, & Lam, 2005). In addition, other comorbidities such as cardiovascular disease, diabetes, and obesity may be present (Ma Elena Jeste et al., 1996; Medina‐Mora et al., 2003; Wingo, Harvey, & Baldessarini, 2009).

A manic episode is defined as the presence of mood shifts characterized by being elevated, expansive, euphoric, or irritable, with increased energy and activity levels, decreased concentration and daily functionality, for at least a week, which can also be associated with psychotic symptoms (First, 2013). In Mexico, BD mania and hypomania have a prevalence of 0.9 and 1.1 per 100,000 people, respectively (Ma Elena Medina‐Mora et al., 2003). Disease prognosis is associated with number of acute episodes, and the risk of having a relapse is higher if the number of previous episodes increases (Ma Elena Medina‐Mora et al., 2003).

Besides the mood and behavioral symptoms, BD is related to cognitive impairment which decreases patient functionality (Jeste et al., 1996). These alterations in cognition are related not only to the acute mood episode but also during euthymia (Wingo et al., 2009). Different traits of impairment in cognitive functions have been described within different mood episodes (Schretlen et al., 2007). For instance, during a depressive episode, the executive functions, such as attentional bias, low mental speed, planning, and working memory are the most affected (Van Der Werf‐eldering, Burger, Holthausen, Aleman, & Nolen, 2010). During a manic episode, lack of impulse control, impaired executive functions (Dixon, Kravariti, Frith, Murray, & McGuire, 2004), attention deficits, (Schretlen et al., 2007) verbal learning impairment, delayed free verbal recall, impaired letter and semantic fluency and decreased speed set‐shifting (Kurtz & Gerraty, 2009) are exhibited. During euthymia, impairment is still noted in executive functioning and present itself in the following areas: working memory, immediate nonverbal memory, problem‐solving, set‐switching tasks, verbal interference, psychomotor and visual scanning speed, visuospatial functioning, auditory, and sustained visual vigilance. The following areas pertaining to speech are also impacted: delayed nonverbal recall, long‐delay verbal free recall, phonemic, and semantic verbal fluency (Kurtz & Gerraty, 2009). The number of acute mood episodes through the subject's life, especially manic episodes, is associated with an increase in cognitive dysfunction (Cipriani, Danti, Carlesi, Cammisuli, & Di Fiorino, 2017).

Lately, new neuroimaging approaches have improved our capacity to understand affective disorders and its relationship to cognition. Studies have discovered a relationship between dysfunction at limbic structures and prefrontal areas in patients with BD (Drevets, 2000; Strakowski, DelBello, Adler, Cecil, & Sax, 2000). Single‐photon emission computerized tomography, SPECT studies have found that high blood perfusion in the striatum and anterior temporal area is correlated with memory impairment and executive dysfunction (Benabarre et al., 2005). Furthermore, increased blood perfusion in the anterior cingulate cortex is related to poor language performance (Benabarre et al., 2005), suggesting a diminished prefrontal modulation of subcortical and medial temporal structures.

However, current studies have not assessed functional neuroimaging and cognition in a longitudinal way, specifically during a manic episode and follow‐up period. This knowledge could lead to a better understanding of the pathophysiology of BD and its relationship with cognitive deficits; this could lead to the development of prognostic and treatment response biomarkers.

The aim in this longitudinal study was to describe brain perfusion patterns and their relationship with cognitive impairment in patients with BD during a manic episode and after 6‐month follow‐up.

## 2 METHODS

### 2.1 Study participants

We included 10 patients with type I BD during a manic episode, diagnosed by an experienced psychiatrist and in accordance with the Diagnostic and Statistical Manual of Mental Disorders (DSM‐IV‐TR) (Association, 2013) criteria and using the South and Central America version of the International Neuropsychiatric Interview (MINI) (Heinze, Sheehan, & Cortés, 2000). The diagnosis for BD needed to be within the past 5 years along with a YMRS score (Young, Biggs, Ziegler, & Meyer, 1978) with at least 20 points. Patients receiving medication to treat BD or electroconvulsive therapy during the 6 months prior to the evaluation were excluded, to avoid confounders in baseline brain perfusion (Miller, Rezai, Alliger, & Andreasen, 1997; Thornton, Schneider, McLean, van Lierop, & Tarzwell, 2014).

Patients were included regardless of gender, socioeconomic status, or ethnicity. We excluded patients with moderate to severe depressive symptoms, Montgomery‐Asberg Depression Rating Scale (MADRS) score (Lobo et al., 2002) with more than 19 points. Subjects with other major psychiatric or neurological conditions including alcohol or substance abuse, and patients with uncontrolled medical conditions were excluded. Pregnant or breastfeeding women were excluded. We used a complete medical history, physical examination and laboratory evaluation (blood count, blood chemistry, hepatic function, thyroid function, urinalysis, and electrocardiogram) to assess exclusion criteria. The sample was obtained from the National Institute of Psychiatry “Ramón de la Fuente Muñiz,” from March 2015 to July 2016.

### 2.2 Assessments

We obtained sociodemographic data and assessed patients with three clinical scales; the YMRS for manic episode severity, the Brief psychiatric rating scale, (BPRS) (Sánchez, Ibáñez, & Pinzón, 2005) which measures clinical severity, evaluating the negative, positive and affective symptoms in psychotic patients, and the MADRS for depression severity (Lobo et al., 2002).

Cognition was measured using the Spanish version of the Screen for Cognitive Impairment in Psychiatry Scale (SCIP‐S), (Pino, Guilera, Rojo, Gómez‐Benito, & Purdon, 2014). This screening tool has five subtests, the Immediate Verbal Learning Test (VLT‐I), the Delay Verbal Learning Test (VLT‐D), the Working Memory Test (WMT), the Verbal Fluency Test (VFT), and the Processing Speed Test (PST). Finally, we assessed brain perfusion with SPECT.

Patients were followed up for 6 months after the manic episode. The SPECT, SCIP‐S, BPRS, MADRS, and YMRS were assessed again.

### 2.3 Neuroimaging protocol

Brain Perfusion SPECT scans were performed at the Department of Neuroimaging at the National Institute of Psychiatry “Ramón de la Fuente Muñiz.” The protocol was performed during resting state using SPECT‐CT dual‐head cameras (PRECEDENCE‐Philips). The regions of interest (ROI) were the following specific Brodmann areas (BA): dorsolateral prefrontal cortex (DLPFC): 9; frontopolar prefrontal cortex (FPC): 10; orbitofrontal cortex (OFC): 11; parietal cortex (PaC): 7; middle temporal gyrus (MTG): 21; superior temporal gyrus (STG): 22; ventral entorhinal cortex (vEC): 28; temporal polar cortex (TP): 38; anterior cingulate cortex (ACC): 24; dorsal anterior cingulate cortex (dACC): 32, subgenual cingulate cortex (sACC): 25; posterior cingulate cortex (PCC): 26; oculomotor area: 8; occipital cortex (OcC) 18; and thalamus bilaterally.

Initially, an intravenous line was started, and then, the participant was placed in a quiet closed room to avoid the external stimulus. After 15 min, 925 MBq of Tc99m‐ethyl cysteine dimer (Neurolite R Accesofarm) was administered with a 40–45 min uptake period. The images were acquired with the shot method, based on the quality standard (Prado & Mena, 1999), obtaining 128 images of 20 s, each one to obtain a total of 5,000,000 counts.

Image processing was made with the Astonish software (Philips), using attenuation correction with CT‐corrected images, and generating axial, coronal and sagittal sections. The data were quantified with the Neurogram software (Segami Corp) using a Talairach map (Goodwin et al., 1997), obtaining volumetric images and voxel to voxel assessment of ROI's.

The data interpretation was performed by a certified nuclear medicine physician based on a quantitative color scale analysis to identify diminished or increased perfusion at ROI´s, using the cerebellum as reference. For the analysis, we calculate the relative perfusion index and the asymmetry index of each ROI using the following formulas:

$$\text{RRI}\;\left(\text{Relative}\;\text{Reuptake}\;\text{Index}\right)=\text{ROI}/\text{Reference}\;\text{ROI}\;\left(\text{cerebellar}\;\text{perfusion}\right).$$

$$\text{AI}\;\left(\text{Asymmetry}\;\text{Index}\right)=\left[\left(\text{right}\;\text{RRI}-\text{left RRI}\right)/\left(\text{right}\;\text{RRI}+\text{left}\;\text{RRI}\right)\right]\times 200.$$

In addition, a semi‐quantification of ROI´s was performed using the voxel to voxel comparative analysis with a control group from a database of age‐matched healthy subjects (Fu et al., 2015) (Neurogam Segamicorp), expressed as a *z*‐score of each ROI.

### 2.4 Statistical analyses

For the descriptive analysis of categorical variables, absolute and relative frequencies were used. For the quantitative variables, means and their respective standard deviations were reported.

We evaluated the data normality distribution through graphical assessment and the Shapiro–Wilk test, and then, we assessed the correlation between the RRI and AI of each bilateral ROI with the following clinical scales: cognition and psychopathology severity at baseline and cognition, psychopathology severity and depressive symptoms at follow‐up.

The correlation between cognition and other clinical scales was evaluated at baseline and follow‐up, separately. We used the Spearman rank correlation test for both analyses. Also, we compared the perfusion of each bilateral ROI, cognition, psychopathology severity, and depression among baseline and follow‐up period using the Wilcoxon rank‐sum test. We performed a correlation analysis between the changes of perfusion with cognition and clinical scales at baseline and follow‐up.

We considered <0.05 as a statistically significant *p*‐value with a confidence interval of 95%. The analysis was performed using Stata software v15.0.

### 2.5 Ethical aspects

This study was conducted according to the guidelines of the Declaration of Helsinki and was approved by the Institutional Ethical Review Board of the National Institute of Psychiatry “Ramón de la Fuente Muñiz.” All participants or legal representative received a study explanation and signed informed consent before entering the study.

## 3 RESULTS

Ten patients were recruited for the study, 8 females and 2 males. The mean age of the sample was 36.3 with an *SD* of 11.3 years old. The rest of the demographic and clinical characteristics are summarized in Table 1. We performed MADRS, YMRS, BPRS, and SCIP‐S, at baseline and 6‐month follow‐up, the summary is in Table 2.

**Table 1:** Demographic and clinical characteristics (*n* = 10)

	Median	Min	Max
Age (years)	36.5	20	58
Sex			
Female, *n*	8		
Male, *n*	2		
Duration of manic episode (weeks)	2	1	8
Time from BD diagnosis (years)	0	0	4
Number of episodes			
Manic, *n*	1	1	2
Depression, *n*	1	0	3
Education (years)	12	9	17.5

**Table 2:** Assessment results during manic episode and follow‐up period

	Manic episode (*n* = 10)	Follow‐up (*n* = 6)	Normal values
	Median (Min–Max)	Median (Min–Max)	
MADRS	4 (0–12)	6 (0–11)	
YMRS	31 (20–56)	2 (0–4)	
BPRS	27 (20–47)	25.5 (21–28)	
SCIP‐S	65 (34–86)	70.5 (45–86)	<70
Immediate verbal learning	15 (7–27)	18 (9–25)	<21
Working memory	18 (9–22)	19 (14–22)	<20
Verbal Fluency	20 (6–28)	19 (12–25)	<19
Delay verbal learning	5 (0–6)	6 (0–10)	<7
Processing speed	8.5 (5–12)	10 (8–14)	<12

Abbreviations: BPRS, Brief Psychiatric Rating Scale; MADRS, Montgomery‐Asberg Depression Rating Scale; SCIP‐S, Screen for Cognitive Impairment in Psychiatry – Spanish version; *SD*, Standard deviation; YMRS, Young Mania Rating Scale.

### 3.1 Comparison with healthy subjects

**Figure 1:**
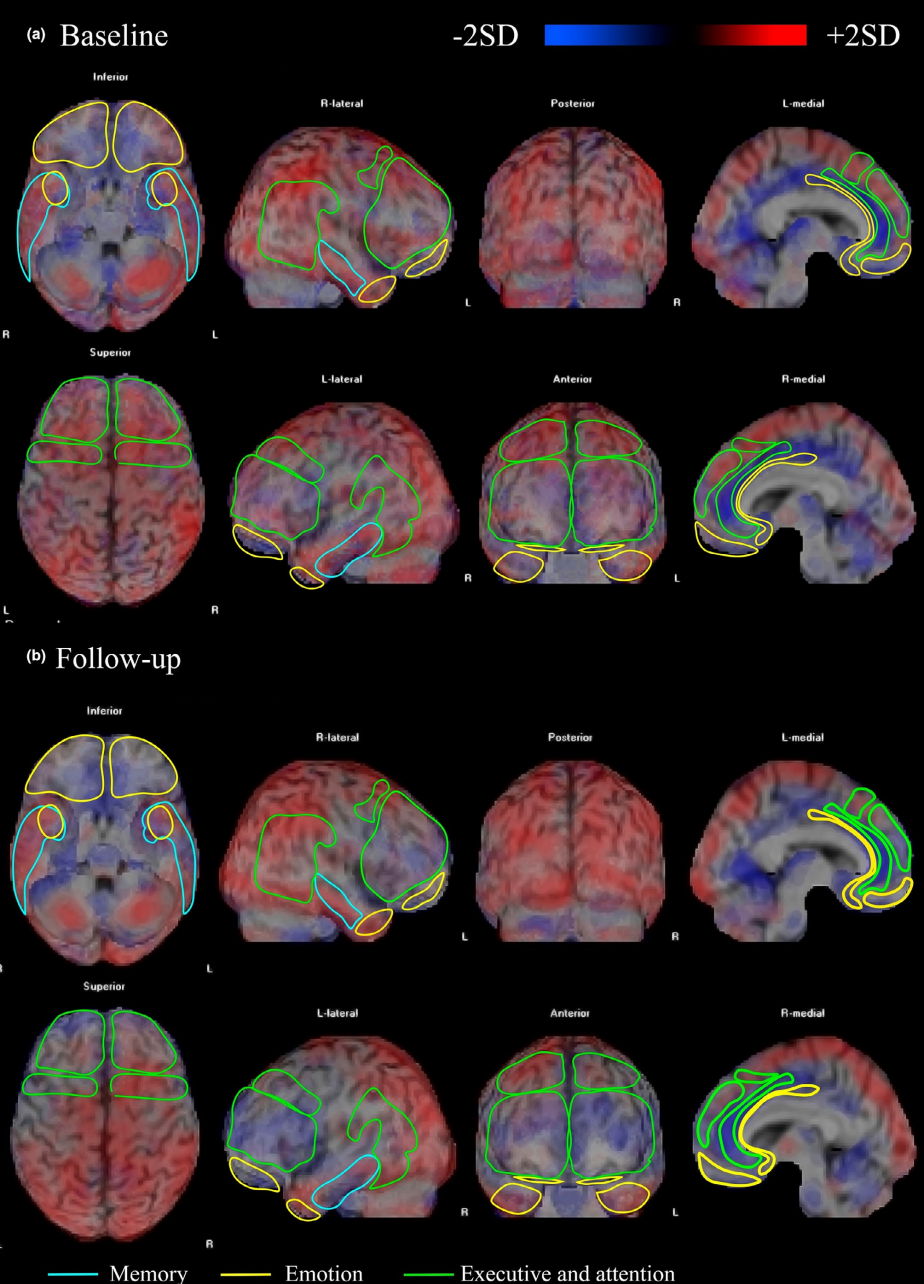
SPECT findings. The following images show anterior, posterior, medial, and lateral cortex of a volumetric image of the brain of patients, in mania (a), and in their 6‐month follow‐up (b). The identification of the color scheme is based on standard deviation of perfusion compared with a group of healthy controls. Noting that blue shows decreased flow in specific cortical areas, and red, cortical hyper perfusion. As well as the gray areas represent no significant changes in relation to the flow of healthy controls. According to the Brodmann Areas, memory includes: 21 (Middle temporal gyrus—MTG), 28 (Ventral entorhinal cortex); emotion network: 11 (Orbitofrontal cortex—OFC), 24 (Anterior cingulate cortex—ACC), 25 (Subgenual cingulate cortex—sACC), 28 (Ventral entorhinal cortex—vEC) and attention and executive networks: 7 (Parietal cortex‐ PaC), 8 (Oculomotor cortex), 9 (Dorsolateral prefrontal cortex—DLPFC), 10 (Frontopolar prefrontal cortex—FPC), 32 (Dorsal anterior cingulate cortex—dACC)

We found no difference among RRI of patients with BD and the healthy subjects' perfusion database, using *z*‐score above or below 2 *SD* at baseline and follow‐up (see Figure 1).

### 3.2 During manic episode

#### 3.2.1 Brain perfusion and clinical scales

We found a positive correlation between the YMRS score and brain perfusion in the right TP (*ρ* = 0.75, *p* = .01). We did not find any correlation between the ROIs, RRI, and BPRS at baseline. Regarding asymmetry, a negative correlation between the BPRS and the AI of dACC (*ρ* = −0.69, *p* = .03), in favor of the right hemisphere was found. There is no correlation among the AI and the YMRS at baseline. We found no correlation with the MADRS and brain perfusion.

#### 3.2.2 Brain perfusion and cognitive assessment

We found a positive correlation between the SCIP‐S total score at baseline with RRI of the right TP (*ρ* = 0.65 *p* = .0435). There was a negative correlation with the right OFC (*ρ* = −0.78 *p* = .0077) and the right sACC (*ρ* = −0.70 *p* = .0256). The correlation of the subscales of the SCIP‐S at baseline and brain perfusion is summarized in Table 3.

**Table 3:** Correlation coefficient between Brodmann areas and ROI from SCIP‐S during manic episode

Brodmann area	Screen for cognitive impairment in psychiatry ‐ Spanish version					
	Immediate verbal learning	Working memory	Verbal fluency	Delay verbal learning	Processing speed	Total score
10 Left					↓ −0.67*	
11 Right		↓ −0.70*			↓ −0.87**	↓ −0.78*
22 Right					↓ −0.69*	
24 Right					↓ −0.82**	
25 Right			↓ −0.74*			↓ −0.70*
32 Right					↓ −0.65*	
38 Right			↑ 0.69*			↑0.64*
38 Left	↑ 0.74 *					
Asymmetry						
Area 21			↑ 0.75*			

Note

Brodmann Area: 10 (FPC), 11 (OFC), 22 (STG), 24 (ACC), 25 (sACC), 32 (dACC), and 38 (TP).

* Significant correlation at *p* < .05 (bilateral).

** Significant correlation at *p* < .01 (Bilateral).

#### 3.2.3 Cognition and the other clinical scales

We found a significant positive correlation between the SCIP‐S total score and the YMRS (*ρ* = 0.65, *p* = .0418). There was no correlation among SCIP‐S subscales and other clinical scales.

### 3.3 Six‐month follow‐up

We were able to assess 6 out of the 10 patients 6 months after the first visit. The rest of the participants were not reachable at the time of the study. They were located far from our center. All participants were euthymic, scoring <2 on the YMRS and <6 on the MADRS.

#### 3.3.1 Brain perfusion and clinical scales

Brain perfusion and clinical scales scores had a positive correlation with the left PaC (*ρ* = 0.8857, *p* = .0188), the left DLPFC (*ρ* = 0.8857, *p* = .0188), the right DLPFC (*ρ* = 0.885, *p* = .0188), and the left FPC (*ρ* = 0.8857, *p* = .018). Asymmetry in perfusion of the sACC was positively correlated with the BPRS score (*ρ* = 0.82, *p* = .0416), in favor of the right hemisphere. No significant correlation among cognition and the other clinical scales was found.

#### 3.3.2 Brain perfusion and cognitive assessment

Six months after the manic episode, the SCIP‐S total score was not significantly correlated with brain perfusion. Regarding the correlation of the subscales of the SCIP‐S and brain perfusion, were summarized in Table 4.

**Table 4:** Correlation coefficient between Brodmann areas and ROI from SCIP‐S after 6 months

Brodmann area	Screen for cognitive impairment in psychiatry – Spanish version					
	Immediate verbal learning	Working memory	Verbal fluency	Delay verbal learning	Processing speed	Total score
9 Left					↓ −0.84*	
9 Right					↓ −0.84*	
10 Left					↓ −0.84*	
21 Right					↓ −0.84*	
22 Right					↓ −0.84*	
24 Left			↓ −0.83*			
24 Right			↓ −0.94**			
32 Left					↓ −0.84*	
32 Right					↓ −0.84*	
38 Left					↓ −0.84*	
Asymmetry						
Area 9			↑ 1.00**			
Area 11					↑ 0.845*	
Area 38				↓ −0.08*		

Note

Brodmann Area: 9 (DLPFC), 10 (FPC), 11 (OFC), 21 (MTG), 22 (STG), 24 (ACC), 32 (dACC), and 38 (TP).

* Significant correlation at *p* < .05 (bilateral).

** Significant correlation at *p* < .01 (Bilateral).

### 3.4 Baseline and Follow‐up comparison

Six months after the maniac episodes, all six patients had a significant reduction in the YMRS (*p* = .001), but no difference in the other clinical scales. The list of patients' medications is summarized in the Table S1.

Compared with baseline, we found a significant RRI reduction in the left DLPFC and left FPC (*p* = .03, *p* = .01, respectively). There was no difference in the perfusion AI across time.

Regarding the longitudinal analysis, we found a negative correlation between the YMRS and the perfusion RRI in the right and left OFC (*ρ* = −0.8827, *p* = .019).

## 4 DISCUSSION

### 4.1 Brain perfusion and clinical outcomes during manic episode

There was no significant difference between healthy subject's perfusion and our participants.

Increased perfusion of the right TP was related to the severity of the manic episodes. Overall, psychiatric symptoms were negatively correlated with the dACC right‐left asymmetry. This finding supports the dysregulation hypothesis which states that there is emotional irregularity corresponding to improper function in the limbic system including the temporal regions (Olson, Plotzker, & Ezzyat, 2007). Previous studies describe a correlation between the right TP and manic rating scores (Migliorelli et al., 1993), as well as with BPRS scale (Bhardwaj, Chakrabarti, Mittal, & Sharan, 2010).

Functional asymmetry at the medial prefrontal cortex has been related to mood disorders. Left alterations are associated with depressive symptom and right side alterations with manic symptoms (Bruder, Stewart, & McGrath, 2017).

### 4.2 Brain perfusion and cognitive performance during manic episode

Cognitive impairment during manic episode correlates with perfusion in the emotional network with increased perfusion in right the OFC and the right sACC and decreased perfusion in the right TP.

Cognitive subscales impairment was associated with perfusion patterns in the following networks:

Emotional network: The Immediate Verbal Learning Test with an increased in perfusion in the left TP; the Working Memory Test with decreased perfusion in the right OFC; and the Verbal Fluency Test with increase perfusion in the right TP and decrease perfusion in the right sACC.
Language network: The Verbal Fluency Test correlates with perfusion asymmetry in the MTG.
Spatial attention network: The Processing Speed test correlates with decreased perfusion in the ACC.

Cognitive impairment during mania seems to be correlated with increased perfusion mainly in the limbic system related areas, which has been associated with the physiopathology of manic episodes, where hyperactivation of limbic structures and hypoactivation of more neocortical regions is present.

Previous fMRI studies have shown a failure to deactivate emotional regulation network areas, such as the medial prefrontal cortex and the TP during cognitive tasks in BD patients during manic episodes (Pomarol‐Clotet et al., 2012). Additionally, decreased activation of the right OFC at rest and decreased activation during word generation in the right FPC were noted (Gabrieli, Poldrack, & Desmond, 1998).

Previous SPECT studies in this population have shown decreased cerebral perfusion in the anterior cortical regions, as well as reduction in the normal anterior‐posterior gradient (Rubin et al., 1995; Silfverskiöld & Risberg, 1989).

### 4.3 Cognitive performance and clinical rating scales association during manic episode

We found a significant positive correlation between total cognition score and the YMRS (*ρ* = 0.65, *p* = .0418). This might be a confounder due to sample size and the high variability in the mean number of years of education (12.55 years) of the participants.

### 4.4 Clinical and cognitive changes at follow‐up

During the 6‐month follow‐up, six patients were in euthymia and receiving different medications not controlled by researchers (see Table S1). Despite the improvement in mania, cognitive deficits remained unchanged.

### 4.5 Brain perfusion and clinical outcomes at follow‐up

Longitudinal analysis showed that right and left OFC increased perfusion correlates with a decrease in manic symptoms, which is in accordance with previous reports (Phillips & Swartz, 2014), and might be explained as a regulatory mechanism from limbic structures. Despite the sample size limitation, this finding might be worth to explore further as possible biomarker associated with clinical improvement.

### 4.6 Brain perfusion and cognitive performance at follow‐up

Cognitive impairment correlated with changes in brain perfusion at the following different functional networks:

Emotional network perfusion: Processing Speed Test with decreased perfusion in the bilateral dACC, the left TP, and correlate positively with the OFC perfusion asymmetry, the Verbal Fluency Test with the increased perfusion in the bilateral ACC, Delay Verbal Learning Test correlate negatively with the TP perfusion asymmetry.
Working memory network: Processing Speed Test with decreased perfusion in the bilateral DLPFC and the left FPC; Verbal Fluency Test correlate positively with the DLPFC perfusion asymmetry.
Language network: Processing Speed Test with perfusion decreased in the right MTG and the right STG.

Another study described similar results of increased perfusion at temporal, frontal and limbic areas in resting state associated with poor executive functions in patients with BD (Benabarre et al., 2005).

Similarly, other authors have found increased perfusion at limbic network‐related areas in euthymic BD type II patients. This has been associated with decreased processing speed when compared to healthy subjects, who had greater activation in parietal areas (Berns, Martin, & Proper, 2002). Another study, using PET scans, euthymic BD patients have shown hypometabolism in prefrontal areas related to verbal fluency test, with no temporal changes (Dye et al., 1999).

Brain perfusion during manic episodes showed prefrontal hypoperfusion, and hyper perfusion at emotional‐related BA which was expected. However, at follow‐up, patients showed overall hypoperfusion and no cognitive improvement even while being in a euthymic state. This might be associated with the patient's medication or related to the BD pathophysiology, suggesting that the time to complete recovery after a manic episode might be longer than 6 months.

### 4.7 Clinical practice significance

At follow‐up, there was a significant reduction in the YMRS scores and overall improvement, and this was related to an increased bilateral perfursion in the OFC.

We did not however find a difference in cognitive performance. This could be related to the patients taking medication, or having time to recover, it is possible that it might take longer than 6 months to improve overall functioning and cognition. Or it also might be the case that cognitive deficits are traits that persist even without affective symptoms, which highlights the importance for the development of new therapeutic approaches that target cognitive function in this group of patients.

### 4.8 Limitations and research recommendations

Some of the limitations of this study are the small sample size, the short follow‐up period and the high attrition rate (40%). Moreover, we could not control the pharmacological treatments after the manic episodes.

Another limitation is that we did not use a method to correct for multiple comparisons so this could increase the chance of inaccurate results.

However, this is one of the few studies that has evaluated brain perfusion and cognition during a manic episode and after a 6‐month follow‐up period, providing insight into understanding the pathophysiology of cognitive impairment in BD.

## 5 CONCLUSIONS

Our study showed that patients presented cognitive impairment and disturbances in brain perfusion during a manic episode. After 6 months, regardless of the patients being in a euthymic state, they did not improve cognitive function and continued to have brain perfusion abnormalities. Large sample sizes studies with longer follow‐up periods are needed to explore the changes in brain perfusion and cognitive function in people with BD.

## CONFLICT OF INTEREST

None to declare.

## AUTHOR CONTRIBUTIONS

All the authors have been involved in the interpretation of the results, drafting of the manuscript, revising critically for important intellectual content; given final approval of the version to be published and agreed to be accountable for all aspects of the work. ME‐G and DG‐M design the study, ME‐G and GA‐O collected the data, ME‐G, KP‐B, and MF‐R performed statistical analysis.

## Supporting information

TableS1

## Data Availability

The data that support the findings of this study are available from the corresponding author upon reasonable request.
