# Supplementary material for: Brain perfusion during manic episode and at 6‐month follow‐up period in bipolar disorder patients: Correlation with cognitive functions
Source: Brain Behav. 2020 May 1;10(6):e01615. doi: 10.1002/brb3.1615 (PMC7303383; doi:10.1002/brb3.1615)
Supplement: Supplementary file 1 — TableS1 [file BRB3-10-e01615-s001.docx]

**Table S1. List of patients´ medication**

|  | **Treatment** |
| --- | --- |
| **Subject 1** | Quetiapine 100mg/ day  Magnesium Valproate 800mg/day |
| **Subject 2** | Haloperidol 7.5mg/day  Biperiden 2mg/day  Valproate Semisodium 1000mg/day |
| **Subject 3** | Quetiapine 225mg/day  Magnesium Valproate 800mg /day |
| **Subject 4** | Magnesium Valproate 1250mg/day  Quetiapine 25mg/day  Paroxetine 15mg/day  Clonazepam 1mg/day |
| **Subject 5** | Quetiapine 300mg/day  Lamotrigine 150mg/day  Clonazepam 0.5mg/day |
| **Subject 6** | Lithium carbonate 900mg/day  Olanzapine 10mg/day |
